# Using Sigma metrics to establish analytical product performance requirements and optimize analytical performance of an *in vitro* diagnostic assay using a theoretical total PSA assay as an example

**DOI:** 10.11613/BM.2018.020903

**Published:** 2018-06-15

**Authors:** Victoria Petrides, Sharon Schneider

**Affiliations:** Abbott, Abbott Park, IL, USA

**Keywords:** total quality management, decision making, goals

## Abstract

**Introduction:**

Establishing analytical performance requirements for in vitro diagnostic (IVD) assays is a challenging process. Manufacturers try to optimize analytical performance by choosing amongst many combinations of different product performance characteristics. Sigma metrics and method decision charts can be helpful aids in choosing appropriate analytical performance requirements. The objective of this research was to demonstrate the use of Sigma metrics and method decision charts to help establish analytical performance requirements and to optimize analytical performance at medical decision concentrations for an IVD assay.

**Materials and methods:**

A range of possible Sigma metrics were determined using three sources for total allowable error (TEa) and hypothetical total PSA assay results. Method decision charts were created for each TEa source and used to identify the maximum precision and bias that the assay could have to maintain sigma level performance of at least 3.

**Results:**

To achieve a sigma performance level of at least 3 for a hypothetical total PSA assay, the maximum allowable coefficient of variation ranged from 5.0% to 11.2% depending on the TEa source. To achieve a sigma performance level of at least 6, the maximum allowable coefficient of variation ranged from 2.5% to 5.6% depending on the TEa source.

**Conclusions:**

Using Sigma metrics and method decision charts when establishing analytical performance requirements can help manufacturers choose product requirements that will optimize IVD assay product performance.

## Introduction

Sigma metrics can be used in many ways: to set quality control rules, to describe the performance of multiple assays using a single measurement procedure, to compare the performance of multiple measurement procedures for a single type of assay, and to describe assay analytical performance for external quality assessment participants (*1*-*5*). Sigma metrics can also be used in a product development setting (*6*).

One of the most challenging tasks for an *in vitro* diagnostic (IVD) assay’s product development team is to agree upon the analytical performance requirements. To an outsider, the task may seem straightforward: identify the intended use and intended users of the product, ask the prospective users for their needs for such an assay, and use the responses to develop the product’s requirements. Often, the needs are high-level: users want an assay that is easy to use, accurate, precise and reliable, but the developer needs to know how accurate and how precise. To answer these questions, one can start by considering how much the assay’s reported result can vary from the true value without impacting the treatment of a patient. In measuring a sample’s concentration, there is inherent uncertainty comprised of a combination of systematic error (*i.e.* bias) and random error (*i.e.* imprecision). This analytical variation is in addition to the uncertainty caused by pre-analytical and biological variation, all three sources of which contribute to overall test result variation (*7*). If the reported analyte concentration is close enough to the true analyte concentration so that the treatment of the patient will be the same, the deviation from the true value can be acceptable. This allowable deviation is commonly referred to as the total error allowable (TEa) and can be expressed in absolute or relative (percentage) units from the true value. A Sigma metrics considers the TEa, bias and precision to provide a single value for assessing the quality of a process based on a single concentration level at a given point in time and is calculated as: sigma = (TEa - |bias|) / precision (*8*). Sigma metrics can be used as an aid to eliminate defects and reduce variability. In the context of a laboratory setting, defects are assay results that could cause a misclassification where the physician suggests an incorrect course of action for a patient. Organizations using Sigma metrics often refer to processes as ranging from a 1-sigma to 6-sigma (or better) process. A 3-sigma assay is generally considered the minimum acceptable performance, whereas as a 6-sigma assay is considered world-class (*9*). The sigma level of an assay can be used to determine the quality control (QC) routine needed for an assay, where higher sigma values require fewer levels of QC material or less frequent QC monitoring. Higher sigma levels mean fewer defects and higher confidence in laboratory results.

The practice of using Sigma metrics to improve and design high quality products has been around for several decades (*6*). One problem IVD manufacturers face in using Sigma metrics, however, is to determine which TEa goal to use during product development since TEa values for many measurands differ greatly, depending on the source. While there is a recommended hierarchy to consider when choosing an appropriate TEa, there is no uniform consensus on which source is most appropriate for a given measurand (*10*).

The objective of this research was to demonstrate the use of Sigma metrics and method decision charts to help establish analytical performance requirements for an IVD assay.

## Materials and methods

Total prostate-specific antigen (PSA) was chosen as an example for establishing analytical performance requirements for multiple reasons. First, there are multiple sources of TEa for total PSA. Second, total PSA has more than one intended use – it may be used for screening subjects for prostate cancer or for monitoring patients (*11*). Lastly, total PSA has generally accepted medical decision concentrations (*11*, *12*).

**Figure 1:**
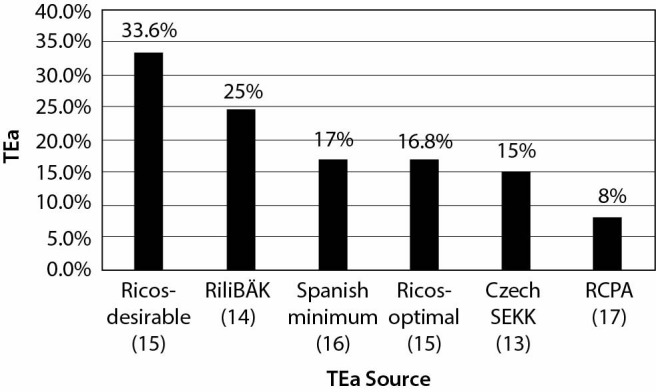
Sources for total allowable error (TEa) specifications used to establish analytical performance requirements for total prostate specific antigen (*13*-*17*)

Six TEa sources, noted in Figure 1, were considered. The European Federation of Clinical Chemistry and Laboratory Medicine (EFLM) published a recommended hierarchy for choosing an appropriate TEa whereby setting required performance specifications are based on three possible models: clinical outcomes, biological variability, and state-of-the-art (*10*). None of the sources based their TEa specifications on clinical outcomes. Two of the sources (Czech Republic external quality assurance program (SEKK) and guidelines of the German medical association for the quality assurance of laboratory medical examinations (RiliBÄK)) are more reflective of specifications based on state-of-the-art (model 3), whereas the remaining four sources are based on biological variability (model 2) (*13*, *14*). It is interesting to note that while Ricos, Spanish minimum and Royal College of Pathologists of Australasia (RCPA) claim to be based on biological variability, there is a four-fold difference in values between the highest and lowest specifications (*15*-*17*). It is not obvious why there is such a large difference but it may be due to the different studies used to determine biological variability. It should be noted, however, that for total PSA, the RCPA TEa is ± 0.4 µg/L up to 5.0 µg/L PSA, and ± 8% > 5.0 µg/L PSA. Similarly, there is a difference of 10% in the two total PSA TEa specifications based on state-of-the-art (25% RiliBÄK TEa *vs* 15% Czech SEKK TEa). These differences illustrate the difficulty manufacturers and laboratories have in deciding which TEa source is most appropriate. Of these six sources, three were used in the assessment: Ricos-desirable, RiliBÄK and Czech SEKK. These three sources were chosen because of their clearly different total PSA TEa values (33.6% for Ricos, 25% for RiliBÄK and 15% for Czech SEKK) and because they represented sources based on biological variability (Ricos) and state-of-the-art (Czech SEKK and RiliBÄK). Furthermore, the Spanish minimum (17%) and Ricos-optimal (16.8%) TEa values were similar to those of Czech SEKK (15%).

A range of possible Sigma metrics were determined using Ricos-desirable, RiliBÄK and Czech SEKK sources for TEa to create a method decision chart using Microsoft Excel 2016 (*18*). These charts were used to identify the maximum precision and bias that the assay could have and still meet specified sigma levels.

Hypothetical values for total PSA precision and bias were given for three formulations (A, B and C) at three concentrations (4.0, 10.0 and 20.0 µg/L), and the Sigma metrics at each concentration was determined using the formula: sigma = (TEa - |bias|) / precision. The sigma values were plotted on a normalized method decision chart so the results could be compared, plotting the percent bias and percent coefficient of variation (CV) as a percentage of TEa (*18*). The two values on the x- and y- axes that were used to create the sigma lines are derived from the formula: sigma = (TEa - |bias|) / precision. Theoretically, if there is 0 imprecision, then the bias can be at the TEa limit. Similarly, if there is 0% bias, then the maximum allowable precision is equal to TEa / sigma. For example, with a TEa limit of 15% and 0% bias, then the imprecision would be 15% CV for a sigma value of 1, 7.5% CV for a sigma value of 2, and so on.

## Results

**Figure 2:**
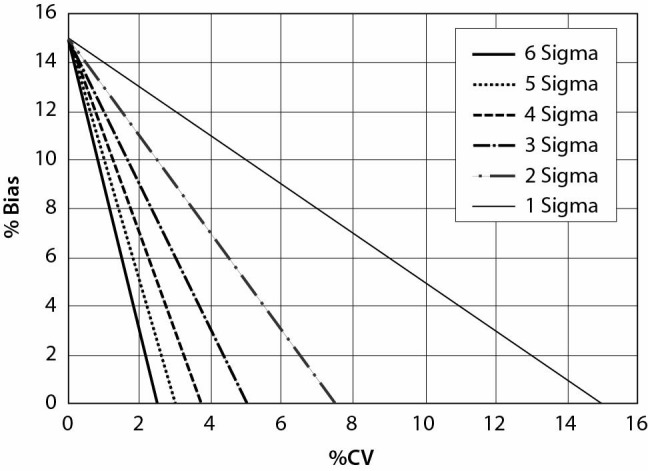
Method decision chart for total prostate specific antigen when using Czech SEKK total allowable error specification of 15% (*13*)

**Figure 3:**
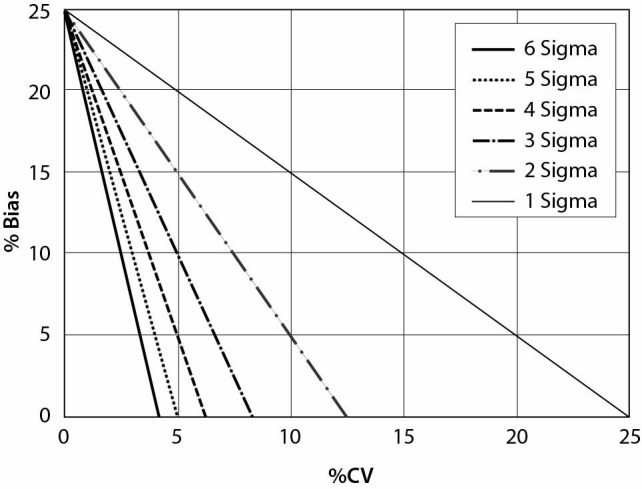
Method decision chart for total prostate specific antigen when using RiliBÄK total allowable error specification of 25% (*14*)

**Figure 4:**
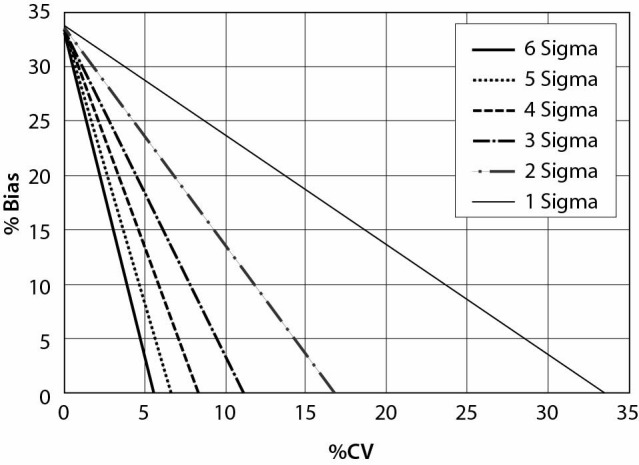
Method decision chart for total prostate specific antigen when using Ricos desirable total allowable error specification of 33.6% (*15*)

To achieve a sigma performance level of at least 6 while assuming zero bias, the maximum allowable CV was 2.5% using Czech SEKK (Figure 2), 4.2% using RiliBÄK (Figure 3), and 5.6% using Ricos-desirable (Figure 4) as TEa sources. The bias theoretically could be up to the same limit as the TEa specification while maintaining a given sigma performance, but only under the unrealistic scenario that the assay has perfect precision (*i.e.* 0%CV). To achieve a sigma performance level of at least 3 while assuming zero bias, the maximum allowable CV was 5.0% using Czech SEKK (Figure 2), 8.3% using RiliBÄK (Figure 3), and 11.2% using Ricos-desirable (Figure 4) as TEa sources.

**Figure 5:**
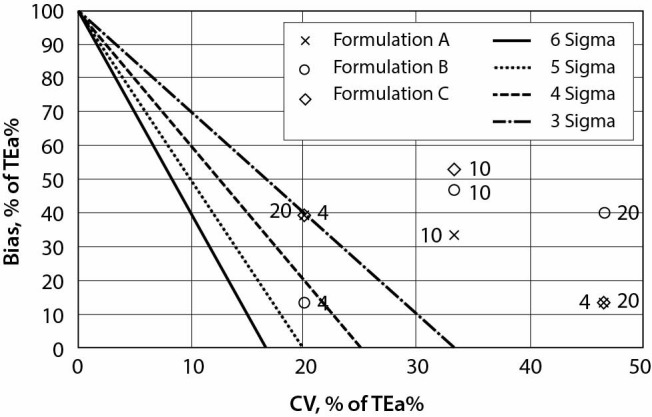
Normalized method decision chart showing sigma values for three hypothetical formulations of a total prostate specific antigen (PSA) assay at three total PSA µg/L concentrations for Czech SEKK total allowable error (TEa) of 15% (*13*)

**Figure 6:**
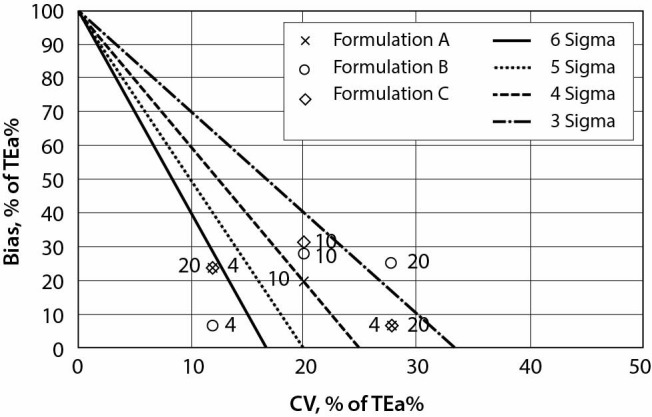
Normalized method decision chart showing sigma values for three hypothetical formulations of a total prostate specific antigen (PSA) assay at three total PSA µg/L concentrations for RiliBÄK total allowable error (TEa) of 25% (*14*)

**Figure 7:**
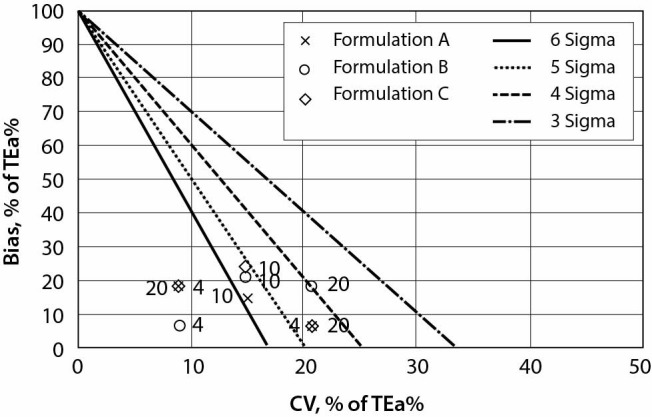
Normalized method decision chart* showing sigma values for three hypothetical formulations of a total prostate specific antigen (PSA) assay at three total PSA µg/L concentrations for Ricos desirable total allowable error (TEa) of 33.6% (*15*)

During the research phase of product development, assay development scientists show the product development team data from feasibility studies. The team will review the data and choose a final formulation with which to proceed for product design verification and validation. Commonly used total PSA medical decision concentrations for prostate cancer screening range from 2.5 to 10.0 µg/L but the medical decision range for monitoring can span from the assay’s lower limit of quantitation to 20.0 µg/L or more (*11*). To illustrate the types of choices a manufacturer might face when choosing an assay formulation, hypothetical precision and bias results were used for three formulations of a total PSA assay (formulations A, B and C) for three samples at different concentrations: 4.0, 10.0 and 20.0 µg/L. The samples at 4.0 and 10.0 µg/L are within the medical decision range of total PSA, while the third sample is above the medical decision range though still within the measuring interval of the hypothetical assay. The results are shown in Table 1 and the Sigma metrics are plotted on a normalized method decision charts in Figures 5, 6 and 7, with each chart showing the sigma value for a different TEa source. Figures 5, 6 and 7 were created using the data shown in Table 1. The %CV and %bias values for each sample concentration for each three assay formulations (A, B, C) were divided by a TEa requirement and plotted on a normalized method decision chart, where the x-axis is the normalized %CV (CV as a percentage of %TEa) and the y-axis is the normalized %Bias (bias as a percentage of %TEa). The numerical labels on each graph identify the hypothetical µg/L concentrations of total PSA samples.

**Table 1:** Hypothetical precision, bias and sigma results for three formulations of a hypothetical total prostate specific antigen assay

				**Sigma values based on specified total allowable error sources**		
Formulation*	**Total PSA** **concentration**	**CV** **(%)**	**Bias** **(%)**	**Ricos desirable** **TEa**	**RiliBÄK** **TEa**	**Czech SEKK** **TEa**
**A**	4 µg/L	7	2	4.5	3.3	1.9
	10 µg/L	5	5	5.7	4.0	2.0
	20 µg/L	3	6	9.2	6.3	3.0
**B**	4 µg/L	3	2	10.5	7.7	4.3
	10 µg/L	5	7	5.3	3.6	1.6
	20 µg/L	7	6	3.9	2.7	1.3
**C**	4 µg/L	3	6	9.2	6.3	3.0
	10 µg/L	5	8	5.1	3.4	1.4
	20 µg/L	7	2	4.5	3.3	1.9
*Formulation refers to different physical properties of the assay, such as different concentrations of proteins, antibodies, detergents, salt, *etc.* Total PSA concentration depicts multiple total PSA values in the analytical measuring interval that are critical for patient management decisions. PSA – prostate specific antigen. CV(%) - coefficient of variation which would typically be derived from a 20-day precision study. Bias(%) - relative bias. TEa - total allowable error. Ricos desirable TEa is 33.6%. RiliBÄK TEa is 25%. RiliBÄK - guidelines of the German medical association for the quality assurance of laboratory medical examinations. Czech SEKK TEa is 15%. SEKK - Czech Republic external quality assurance program.						

Given the hypothetical data provided in the Table 1 and the normalized method decision chart in Figures 5, 6 and 7, an assay development team would try to determine which of the three hypothetical total PSA formulations could achieve the highest quality performance. None of the formulations reflect 6 sigma quality across all samples using any of the TEa specifications; however, the sample at 20.0 µg/L using formulation A and the sample at 4.0 µg/L using formulations B and C had sigma values greater than 6 when using Ricos desirable or RiliBÄK TEa specifications. Since the sample at 20.0 µg/L is not within the medical decision range but the sample at 4.0 µg/L is, the assay development team would likely place greater value on having higher quality performance on the sample at 4.0 µg/L.

From Figures 2 – 4 one can see that the maximum CV required to achieve the minimum acceptable sigma of 3 is 5.0%, 8.3% or 11.2% when assuming zero bias, depending on the TEa source. From Table 1, laboratories using Czech SEKK TEa values could only achieve 3 sigma performance on one sample from each formulation, suggesting that this assay might not be marketable to those customers. Laboratories using RiliBÄK TEa values could achieve 6 sigma performance for one sample and at least 3 sigma performance for two samples using formulations A and C, but did not perform as well using formulation B. This suggests that the team may want to rule out formulation B. The Sigma metrics using the Ricos desirable TEa demonstrate that the assay can achieve at least 4 sigma performances on formulations A and C. Since the performance at medical decision concentrations of 4.0 µg/L and 10.0 µg/L is more critical than the performance at 20.0 µg/L and formulation C has higher sigma performance at 4.0 µg/L, formulation C appears to be the best choice in maintaining the highest quality performance amongst the three options.

## Discussion

With a 3-sigma assay being generally considered the minimum acceptable performance and a 6-sigma assay performance considered world-class, Sigma metrics and method decision charts can be used to help determine the optimal formulation for a product as illustrated with the hypothetical total PSA example (*9*). Additionally, the example showed that Sigma metrics and method decision charts could be used to determine the upper limits of the bias and precision values that should be considered during the development of an IVD product. Furthermore, manufacturers can create different method decision charts for different total error requirements. While a manufacturer’s assay may be developed with a target market in mind, and the manufacturer might assume most laboratories within the target market would use a specific TEa source, understanding how laboratories that use a different TEa source can aid the manufacturer in advising the laboratory. This information can also assist the laboratory in knowing the kind of performance to expect.

Another point to consider when using Sigma metrics is that bias and precision influence the Sigma metrics differently, with precision having a greater impact. Petersen and Klee elaborate on this topic 2014 in their paper (*19*).

This exercise used total PSA as an example of how performance specifications for bias and precision could be set. It should be noted that there is not a certified reference material for total PSA and thus the bias aspect of the analyte could only be estimated relative to a stated total PSA method or material. While the bias for total PSA is not a bias in the sense that it is estimated against a universally recognized true value, this exercise was meant to demonstrate how Sigma metrics could be used during product development and the analyte is an example meant to illustrate the concept.

There are several limitations with this approach, one of the most obvious being that there are many other factors besides precision, bias and sigma performance must be considered when making decisions during product development. The example is intentionally over-simplified to highlight the key details in the decision-making process. During the actual product development process, many other factors including stability, manufacturability, cost, *etc.*, must be considered when choosing amongst various product formulations. Another limitation is that not all laboratories use Sigma metrics or TEa specifications, so even if the manufacturer develops a product with sigma performance in mind, the optimization may not translate to the needs of those laboratories. Additionally, any estimate of sigma performance is just a snapshot in time and can vary across different concentrations of the measurand. Despite such limitations, designing products with high quality performance in mind up front will still result in better quality in the long run.

In conclusion, using Sigma metrics and method decision charts when establishing analytical performance requirements can help manufacturers choose requirements that will optimize IVD assay product performance.
